# Complete chloroplast genome sequence of *Chrysosplenium sinicum* and *Chrysosplenium lanuginosum* (Saxifragaceae)

**DOI:** 10.1080/23802359.2019.1623099

**Published:** 2019-07-10

**Authors:** Rui Liao, Xiang Dong, Zhi-Hua Wu, Rui Qin, Hong Liu

**Affiliations:** a Key Laboratory for Protection and Application of Special Plant Germplasm in Wuling Area of Hubei Province, South-Central University for Nationalities, Wuhan, China;; b College of Life Science, South-Central University for Nationalities, Wuhan, China

**Keywords:** Chrysosplenium, chloroplast genomes, phylogeny

## Abstract

The complete chloroplast genome of *Chrysosplenium sinicum* Maxim and *Chrysosplenium lanuginosum* Hook. f. et Thoms. were reported in this study. The chloroplast genomes were 152,524 bp and 151,512 bp for *C. sinicum* and *C. lanuginosum*, respectively. LSC and SSC of 83,330 bp and 18,018 bp were separated by two IRs of 25,588 bp each in *C. sinicum*. While *C. lanuginosum* contained IRs of 25,985 bp, LSC of 82,250 bp and SSC of 17,292 bp, for a total 151,512 bp length. The chloroplast genomes both contained 117 genes, including 82 protein-coding genes, 31 tRNA genes, and four rRNA genes.

*Chrysosplenium* L. (Saxifragaceae) comprises about 65–70 species worldwide, mainly distributed in the northern hemisphere, with high diversity in East Asia and only two species in the southern hemispheric (Hara 1957; Soltis et al. 2001; Lan et al. 2018, 2019). The species of *Chrysosplenium* were usually treated as traditional Chinese herbal medicines for significant pharmacological effects and the flavonols component (Qin et al. 2018). In this study, we reported the chloroplast genome of *Chrysosplenium sinicum* and *Chrysosplenium lanuginosum*. With these data, we reconstructed the phylogenetic tree to confirm the relationship within Dipsacales and Rosids, which will support useful information for the further study of the genus.

The materials of *C. sinicum* (TTZ2016060806646, N 31°07′44″, E 115°46′53″) and *C. lanuginosum* (HSG2017041607384, N 31°31′08″, E 116°09′26″) were collected from Jin-Zhai county, An-Hui, China, the voucher specimens were deposited at Herbarium of South-Central University for Nationalities (HSN). The total genomic DNA was extracted using a modified cetyltrimethylammonium bromide (CTAB) method (Doyle 1991) and sequenced using the Illumina platform at Novogene Company (Beijing, China). After filtered the low-quality data and adaptors, the obtained clean data were aligned to *Bergenia scopulosa* (NC_036061.1) with bwa-0.7.12 (Li and Durbin 2010). The aligned reads were then assembled with ABYSS-2.0.2 after the best Kmer was chosen with kmergenie (Chikhi and Medvedev 2014). Then, connected the overlap and scaffolding again by SSPACE_Standard_v3.0 to get the final scaffolds. Finally, we mapped the scaffolds to the reference to find the IR region and annotated using DOGMA.

The complete chloroplast genome of C. *sinicum* was 152,524 bp in length (MK814606) and composed of two inverted repeats (IRs) of 25,588 bp which divide LSC of 83,330 bp and SSC of 18,018 bp, the average GC content was 37.32%. On the other hand, the complete chloroplast genome size of C. *lanuginosum* was 151,512 bp in length (MK814607), the average GC content was 37.66%. Separating LSC of 82,250 bp and SSC of 17,292 bp, a pair of IRs was 25,985 bp long in each. The plastid genomes both encoded 117 functional genes, including 82 protein-coding genes, 31 tRNA genes, and four rRNA genes.

Phylogenetic analysis was performed using whole chloroplast coding sequences of C. *sinicum* and C. *lanuginosum*, combined with 10 species of Saxifragales, 13 species of Dipsacales, Vitales, Rosales, Fabales, and two Haloxylon species as the outgroups. The phylogenetic relationships were reconstructed by means of maximum-likelihood (ML), Bayesian inference (BI) and neighbour-joining (NJ) with the model of GTR + G + I. According to the morphology of flowers, *Chrysosplenium* appears to be closely related to Dipsacales (Wu 1981). However, the phylogenetic evidence with three combined methods consistently supported that *Chrysosplenium* is more related to Saxifragaceae than to Dipsacales with high confidence (Figure 1). The similarity between *Chrysosplenium* and Dipsacales may be the result of convergent evolution due to similar habitats. In addition, Saxifragales was sister to Vitales and Rosales in Angiosperm Phylogeny Group (APG) IV which is still having some controversy (Moore et al. 2010). However, it would be necessary to resolve the relationship with more molecular samples.

**Figure 1.**
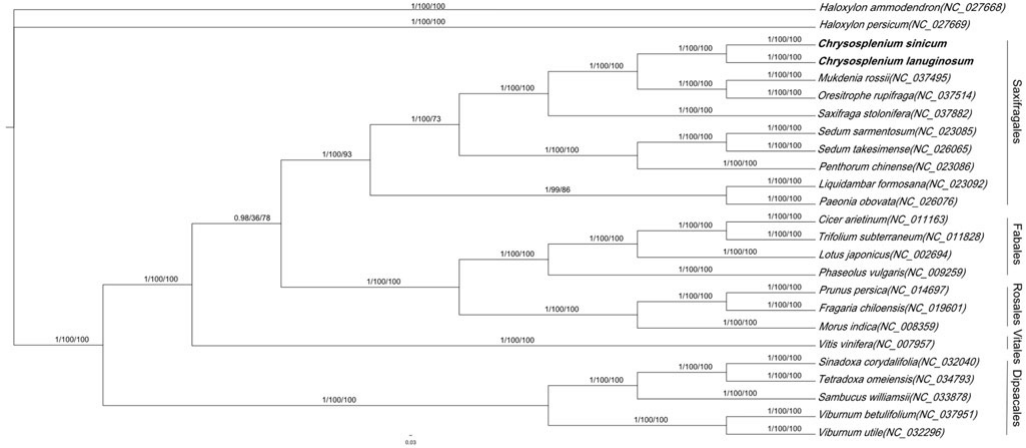
Phylogenetic tree reconstructed by Maximum-likelihood (ML) analysis based on whole chloroplast genome sequences from 25 species. The values on each node represent the posterior probabilities from Bayesian inference (left), the bootstrap value from maximum-likelihood (middle) and neighbour-joining (right), separately.
